# The Fire Ant Social Chromosome Exerts a Major Influence on Genome Regulation

**DOI:** 10.1093/molbev/msaf112

**Published:** 2025-05-24

**Authors:** Beryl M Jones, Alex H Waugh, Michael A Catto, Sasha Kay, Karl M Glastad, Michael A D Goodisman, Sarah D Kocher, Brendan G Hunt

**Affiliations:** Department of Entomology, University of Kentucky, Lexington, KY 40508, USA; Department of Genetics, University of Georgia, Athens, GA 30602, USA; School of Biological Sciences, Georgia Institute of Technology, Atlanta, GA 30318, USA; Department of Entomology, University of Georgia, Griffin, GA 30223, USA; Department of Biology, University of Rochester, Rochester, NY 14620, USA; School of Biological Sciences, Georgia Institute of Technology, Atlanta, GA 30318, USA; Department of Ecology and Evolutionary Biology, Lewis-Sigler Institute for Integrative Genomics, Princeton University, Princeton, NJ 08544, USA; Department of Genetics, University of Georgia, Athens, GA 30602, USA; Department of Entomology, University of Georgia, Griffin, GA 30223, USA

**Keywords:** supergenes, gene regulation, evolution, epigenetics, trait polymorphism

## Abstract

Supergenes underlying complex trait polymorphisms ensure that sets of coadapted alleles remain genetically linked. Despite their prevalence in nature, the mechanisms of supergene effects on genome regulation are poorly understood. In the fire ant *Solenopsis invicta*, a supergene containing over 500 individual genes influences trait variation in multiple castes to collectively underpin a colony level social polymorphism. Here, we present results of an integrative investigation of supergene effects on gene regulation. We present analyses of ATAC-seq data to investigate variation in chromatin accessibility by supergene genotype and STARR-seq data to characterize enhancer activity by supergene haplotype. Integration with gene co-expression analyses, newly mapped intact transposable elements (TEs), and previously identified copy number variants (CNVs) collectively reveals widespread effects of the supergene on chromatin structure, gene transcription, and regulatory element activity, with a genome-wide bias for open chromatin and increased expression in the presence of the derived supergene haplotype, particularly in regions that harbor intact TEs. Integrated consideration of CNVs and regulatory element divergence suggests each evolved in concert to shape the expression of supergene encoded factors, including several transcription factors that may directly contribute to the trans-regulatory footprint of a heteromorphic social chromosome. Overall, we show how genome structure in the form of a supergene has wide-reaching effects on gene regulation and gene expression.

## Introduction

Genome structure can have fundamentally important effects on evolution and adaptation. For example, chromosomal inversions can facilitate the evolution of complex trait polymorphisms such as ecotypes (Todesco et al. 2020; Hager et al. 2022; Matschiner et al. 2022), mating morphs (Mank 2023), and sexes (Branco et al. 2018; Duhamel et al. 2022) by ensuring sets of coadapted alleles remain genetically linked (Dobzhansky and Epling 1948) in what is known as a supergene (Schwander et al. 2014; Thompson and Jiggins 2014). Supergenes have been documented across diverse eukaryotic taxa (Wellenreuther and Bernatchez 2018), and chromosomal inversion polymorphisms are pervasive in natural populations (Dobzhansky and Sturtevant 1938; Huang et al. 2014; Todesco et al. 2020; Harringmeyer and Hoekstra 2022). Despite this prevalence, how supergenes influence genome function beyond limiting recombination and affecting selection on linked regions is not well understood.

Among ants, at least 5 independent origins of supergenes are known to be associated with polymorphic life histories and social forms (Kay et al. 2022; Chapuisat 2023; Lajmi et al. 2024). In the red imported fire ant, *Solenopsis invicta*, distinct supergene genotypes control whether a colony contains a single egg-laying queen (monogyne social form) or multiple egg-laying queens (polygyne social form) (Ross and Keller 1998; Wang et al. 2013; Yan et al. 2020). This variation in colony social organization arises in conjunction with pronounced supergene effects on caste determination (Buechel et al. 2014), reproductive maturation (DeHeer 2002; Nipitwattanaphon et al. 2013; Waugh et al. 2024), dispersal (DeHeer et al. 1999; Goodisman et al. 2000), male survival (Hettesheimer et al. 2025), and queen acceptance by workers (Keller and Ross 1998; Ross and Keller 2002; Zeng et al. 2022). Monogyne queens invariably are homozygous for the *Social B* (*SB*) supergene haplotype, and their colonies reproduce when new queens independently disperse (Ross and Keller 1998; DeHeer et al. 1999; DeHeer 2002; Wang et al. 2013; Yan et al. 2020). Polygyne reproductive queens are heterozygous for *SB* and the chromosomal inversion containing *Sb* supergene alleles, and their colonies reproduce by budding, with workers always present to support the queen (Vargo and Porter 1989; Ross and Keller 1998; DeHeer et al. 1999; DeHeer 2002; Wang et al. 2013; Yan et al. 2020).

The *Sb* haplotype in fire ants is formed by 3 linked chromosomal inversions and a linked centromeric region of suppressed recombination that collectively comprise a single supergene on a social chromosome (Wang et al. 2013; Yan et al. 2020). The *Sb* chromosome variant spread by introgression to several *Solenopsis* species within the last million years (Helleu et al. 2022; Stolle et al. 2022), where it underpins polygynous social organization (Yan et al. 2020). The supergene region of the social chromosome contains over 500 individual genes (Wang et al. 2013; Yan et al. 2020) and prior research has revealed genetic differentiation of the *SB* and *Sb* variants, including the presence of nonsynonymous substitutions (Pracana et al. 2017; Cohanim et al. 2018; Martinez-Ruiz et al. 2020). Since its origin, *Sb* has also increased in length by over 30% (Stolle et al. 2019), an expansion that coincides with extensive social chromosome-linked copy number variation in transposable elements (TEs) and typical (host) genes (Pracana et al. 2017; Cohanim et al. 2018; Fontana et al. 2020). Whether and how these TEs and gene copies lead to the striking phenotypic differences between social forms of the fire ant is still largely unknown.

We used a combination of genomic approaches to investigate supergene effects on gene regulation in *S. invicta*. We generated ATAC-seq data from individual worker brains to profile chromatin accessibility, leveraged multiple existing RNA-seq data sets (Chandra et al. 2018; Arsenault et al. 2020, 2023) to identify gene expression modules linked to supergene genotype, mapped intact TEs and known copy number variants (CNVs) (Fontana et al. 2020) to long-read genome assemblies (Yan et al. 2020; Helleu et al. 2022), and generated STARR-seq data to characterize enhancer activity of *SB* and *Sb* DNA fragments. Our integrative analyses reveal widespread supergene effects on chromatin remodeling, gene transcription, and regulatory element activity that are heavily biased toward *Sb* activation and often coincide with genomic regions harboring intact TEs. Our results further suggest structural variants and regulatory elements evolved in concert to shape the expression of supergene encoded factors, including several transcription factors (TF) we identify as potential drivers of the trans-regulatory footprint of the social chromosome.

## Results and Discussion

### The Supergene Affects Global Patterns of Transcription

**Fig. 1.**
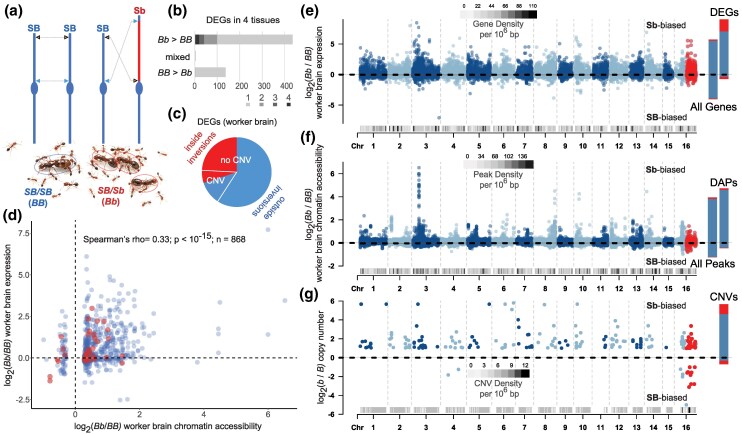
Gene regulatory and copy number variation attributable to the fire ant social chromosome. a) The derived structural variant of chromosome 16 (*Sb*; inversions shown as red chromosomal region) (Helleu et al. 2022) is restricted to the polygyne social form (having multiple reproductive queens), as opposed to the monogyne social form, of *S. invicta*. Polygyne reproductive queens are heterozygotes for *Sb* and the ancestral structural variant (*SB*), while their offspring are a mix of genotypes (*Sb* homozygotes are scarce). Photos courtesy of Haolin Zeng. b) Counts of DEGs according to RNA-seq between *SB/SB* (*BB*) and *SB/Sb* (*Bb*) in worker brains and gasters and alate gyne (queen) brains and ovaries show bias toward upregulation in *SB/Sb* (*n* = 6 to 8 replicates per pairwise comparison; supplementary table S1, Supplementary Material online). c) Proportion of DEGs in worker brains mapped inside or outside the supergene inversion region and with or without *Sb* versus *SB* increased CNVs shows most DEGs map outside the supergene inversions and lack supergene CNVs. d) Scatterplot of fold difference by supergene genotype in significant DAPs according to ATAC-seq and corresponding RNA-seq fold difference in expression, each from worker brains, shows a significant positive correlation between gene expression and chromatin accessibility with a skew toward increased accessibility and expression in *SB/Sb* relative to *SB/SB*. Genes mapped to the *SB* homologous region of the *Sb* supergene inversions are shown in red. e) log2(*SB/Sb*/*SB/SB*) gene expression level by chromosome position. f) log2(*SB/Sb*/*SB/SB*) chromatin accessibility peaks by chromosome position. g) log2(*Sb/SB*) copy number for CNVs (Fontana et al. 2020) by chromosome position mapped to *SB*. For e) to g), red dots map to the supergene inversions, grayscale tracks along *x* axes show feature density by position, and dashed lines represent a fold-change value of 0.

To provide insight into the gene regulatory effects of the polygyne-affiliated *Sb* variant of the fire ant supergene on the social chromosome (chromosome 16; Fig. 1a), we first reanalyzed published RNA-seq data (supplementary table S1, Supplementary Material online). Pairwise comparisons of *SB/Sb* and *SB/SB* gene expression data from worker brains, worker gasters, alate gyne (queen) brains, and queen ovaries (*n* = 6 to 8 biological replicates per group; supplementary table S1, Supplementary Material online) illustrate a striking bias among differentially expressed genes (DEGs) toward upregulation in *SB/Sb* relative to *SB/SB* samples (Fig. 1b; supplementary table S2, Supplementary Material online) (Arsenault et al. 2023); *Sb/Sb* individuals are low fitness and were excluded from this analysis (Hallar et al. 2007). A minority of observed DEGs mapped within the supergene region or exhibited copy number differences between *Sb* and *SB* variants of the social chromosome (Fig. 1c). This pattern is consistent with substantial trans-regulatory effects of the supergene (Arsenault et al. 2020).

To assess variation in genome-wide patterns of chromatin accessibility by supergene genotype, we generated ATAC-seq (Buenrostro et al. 2013, 2015) data from individual *SB/Sb* and *SB/SB* worker brains (*n* = 5 biological replicates; supplementary table S3, Supplementary Material online). Our ATAC-seq data revealed that the increase in gene activity in *SB/Sb* (relative to *SB/SB*) is reflected by changes in chromatin accessibility. We observed over 10-fold more significant differentially accessible peaks (DAPs) with higher accessibility in *SB/Sb* than in *SB/SB* (supplementary table S4, Supplementary Material online). We found that DAPs were enriched for motifs of the TFs DNA replication-related element factor (Dref), Boundary element-associated factory of 32kD (BEAF-32), Erect wing (Ewg), and Pannier (Pnr) with Benjamini–corrected q-value < 0.1 (supplementary table S5, Supplementary Material online). These TFs are not located within the supergene, suggesting the enrichment of these TF motifs in DAPs is a downstream consequence of trans-regulating factors within the inversion (note that the ortholog of BEAF-32 is unknown in *S. invicta*, but the other TFs are annotated and not within the inverted region). Dref and BEAF-32 are known to physically interact in *Drosophila* and play a role in chromatin organization, while both Ewg and Pnr are involved in nervous system development and synaptic growth, among other functions (Jenkins et al. 2022). Gene Ontology (GO) annotations of genes near DAPs (937 genes assigned to DAPs based on proximity, see Methods) were enriched for 3 biological processes and 2 molecular functions: “fatty acid biosynthetic process”, “monocarboxylic acid biosynthetic process”, “DNA integration”, “3-oxoacyl-[acyl-carrier-protein] synthase activity”, and “fatty acid synthase activity”, respectively (FDR < 0.1; supplementary table S6, Supplementary Material online). Variation in the regulation of genes that function in fatty acid biosynthesis could potentially contribute to known differences in fat accumulation by gynes (Keller and Ross 1993; Waugh et al. 2024) and variation in the cuticular hydrocarbon profiles of mature queens (Keller and Ross 1998; Zeng et al. 2022) by supergene genotype in *S. invicta*. In this manner, genes involved in fatty acid biosynthesis represent intriguing candidates for pleiotropic effects on trait variation relevant to both nutrient reserve formation and chemical communication.

We observed a highly significant positive correlation between genotypic effects on chromatin accessibility and gene expression levels in worker brains based on an assessment of the 868 DAPs proximal to genes with expression data in the RNA-seq meta-analysis (Fig. 1d). Global visualizations of RNA-seq (Fig. 1e) and ATAC-seq (Fig. 1f) fold-change values between *SB/Sb* and *SB/SB* illustrate a genome-wide distribution of loci with increased gene activity and chromatin accessibility in *SB/Sb* samples. Because many genes throughout the genome exhibit increased copy number in the *Sb* structural variant, apparently due to TE-mediated translocation into and/or duplication within the supergene region of *Sb* (Stolle et al. 2019; Fontana et al. 2020; CNVs; Fig. 1g; supplementary table S7, Supplementary Material online), we hypothesize that some of these supergene-linked CNVs make contributions to the trans-regulatory effects of the relatively young *Sb* structural variant.

### Constitutive Supergene Effects Are Reflected by a Small Co-expression Module with Genes from Multiple Chromosomes

**Fig. 2.**
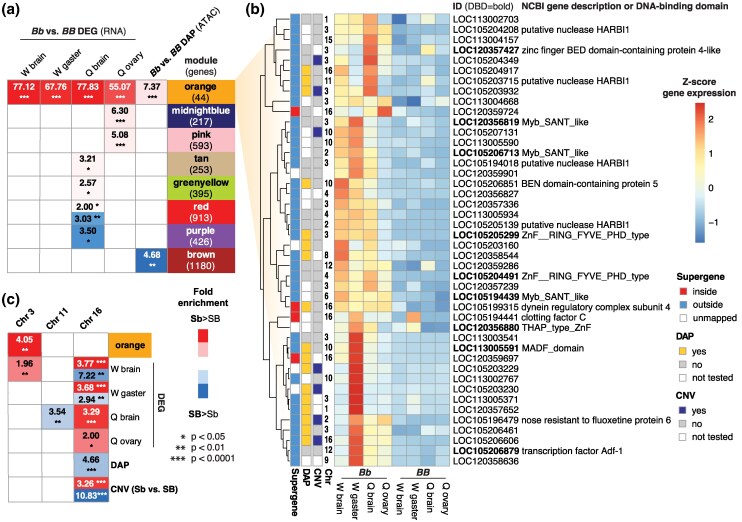
Gene co-expression modules and chromosomes enriched for differential activity by social chromosome genotype. a) WGCNA modules with significant enrichment of DEGs or genes with proximal DAPs. Fold enrichment values are shown relative to expected frequency based on module size, with significance indicated for a hypergeometric overlap test. W, worker; Q, alate gyne (queen). b) Heatmap of standardized expression for genes belonging to the orange gene co-expression module with gene descriptions or included DNA-binding domains. c) Chromosomes with significant enrichment of DEGs, genes with proximal DAPs, or CNVs by supergene genotype. Fold enrichment values are shown with significance relative to expected frequency based on chromosome gene number indicated for a hypergeometric overlap test. All fold enrichment and *P*-values are provided in supplementary tables S9 and S11, Supplementary Material online.

To further characterize the loci differentially regulated by social chromosome genotype, we performed a weighted gene co-expression analysis (Langfelder and Horvath 2008). We included information from 122 RNA-seq libraries (supplementary table S1, Supplementary Material online) including *SB/SB*, *SB/Sb*, and *Sb/Sb* samples of individual queen and worker tissues from both monogyne and polygyne colonies (Chandra et al. 2018; Arsenault et al. 2020, 2023). This resulted in the assignment of 26 gene co-expression modules (supplementary table S8, Supplementary Material online), each of which we examined for enrichment of social chromosome genotype-affiliated pairwise differential expression, chromatin accessibility, and GO enrichment (Fig. 2a; supplementary tables S9 and S10, Supplementary Material online).

The smallest gene co-expression module assigned, orange, contains only 44 genes but is highly enriched for DEGs in all 4 of our focal pairwise RNA-seq comparisons between *SB/Sb* and *SB/SB* (Fig. 2a), containing 19 of 20 4-way overlapping DEGs. Thirty-three of 44 genes from orange are significant DEGs in worker brain, with all 33 of those exhibiting higher expression in *SB/Sb* versus *SB/SB* samples. Orange is also significantly enriched for DAPs between *SB/Sb* and *SB/SB* worker brains (Fig. 2a). Only 7 of the 36 orange module genes with CNV data (Fontana et al. 2020) exhibit supergene-linked copy number variation (Fig. 2b), highlighting that a minority of loci with constitutive expression responsiveness to *Sb* are supergene-linked CNVs. Thus, constitutive expression effects of *Sb* are usually not the result of a simple dosage effect of supergene-linked copy number variation (Fontana et al. 2020). Instead, our results suggest constitutive changes in expression may most commonly be caused by trans-regulatory links between supergene encoded factors and genes residing on other chromosomes. Among the 44 genes in orange, 6 are mapped to chromosome 16, the supergene-containing social chromosome, 5 are located on unplaced scaffolds that potentially belong to chromosome 16, and 13 are mapped to chromosome 3, which is devoid of polygyne-affiliated structural variants (Yan et al. 2020) (Fig. 2b). Chromosome 3, like chromosome 16, is significantly enriched for worker brain DEGs. Moreover, chromosome 3 is the only chromosome exhibiting significant enrichment of genes belonging to the orange module following multiple test correction (Fig. 2c; supplementary table S11, Supplementary Material online). The fact that the constitutively *Sb*-responsive orange co-expression module is not enriched for genes mapped to the social chromosome but is enriched for genes mapped to a different chromosome illustrates that the *Sb* supergene haplotype does exhibit strong trans-regulatory links to the remainder of the genome.

### Genomic Regions with High Intact TE Density May Be Hotspots of Social Chromosome Regulatory Effects

Transposons can give rise to new genes through “molecular domestication” (Volff 2006). The orange co-expression module includes 4 distinct (non-CNV) copies of genes encoding the transposon derived protein HARBI1, none of which map to chromosome 16 (Fig. 2b). In a vertebrate study, HARBI1 and a Myb-like protein (including a SANT/Myb/trihelix domain that binds to DNA) were both shown to be derived from a single *Harbinger* transposase (Sinzelle et al. 2008). Remarkably, 3 distinct (non-CNV) uncharacterized proteins with Myb_SANT_like DNA binding domains (Pfam PF13837 or InterPro IPR028002), none of which map to chromosome 16, are present among the 44 orange module genes. This suggests that the expression of multiple HARBI1 and Myb-like proteins is subject to coordinated activation by *Sb* encoded factors. The SANT/Myb/trihelix motif has also been found to function as a DNA binding domain for many TFs (Kapitonov and Jurka 2004), which may play a role in this activation.

Based on the domesticated transposons belonging to the supergene responsive orange co-expression module and the importance of TEs to the formation (Yan et al. 2020) and size expansion (Stolle et al. 2019; Fontana et al. 2020) of *Sb*, we were interested in investigating whether variation in the TE landscape intersected with regional variation in the gene regulatory landscape by supergene genotype. TF binding sites are frequently embedded within TEs (Sundaram et al. 2014), and TEs may be a frequent source of gene regulatory network innovations (Feschotte 2008; Chuong et al. 2017). Thus, we mapped structurally intact TEs (Ou et al. 2019) in the genome of *S. invicta* to assess whether proximity to TEs is related to whether chromatin exhibits differential accessibility and genes exhibit differential expression by supergene genotype. We observed that intact TE density is positively correlated with supergene-differentiated DAPs, CNVs, and DEGs across the genome. This relationship is strongest for DAPs and in each case is strongest when considering large, 1 Mb genomic windows (Table 1).

**Table 1:** Kendall's rank correlation coefficients (tau) between intact TE counts and supergene associated regulation or CNVs, with partial correlations controlling for gene counts

Variable (*X*)	Correlation tau *_X,_* _TE count_	Partial correlation tau *_X,_* _TE count | gene count_
1 Mb windows		
DEG count^a^	0.415^e^	0.266^e^
DAP count^b^	0.572^e^	0.483^e^
CNV count^c^	0.387^e^	0.329^e^
100 kb windows		
DEG count	0.027	0.020
DAP count	0.160^e^	0.157^e^
CNV count	0.108^d^	0.106^e^

^a^Counted as DEG if 1 or more of 4 focal comparisons (Fig. 1b) is significant.

^b^All ATAC-seq DAPs between *Bb* and *BB* worker brains are reported, regardless of proximity to genes.

^c^CNVs from Fontana et al. (2020) mapped to long-read genome builds.

^d^ *P* < 10^−10^.

^e^ *P* < 10^−15^.

**Fig. 3.**
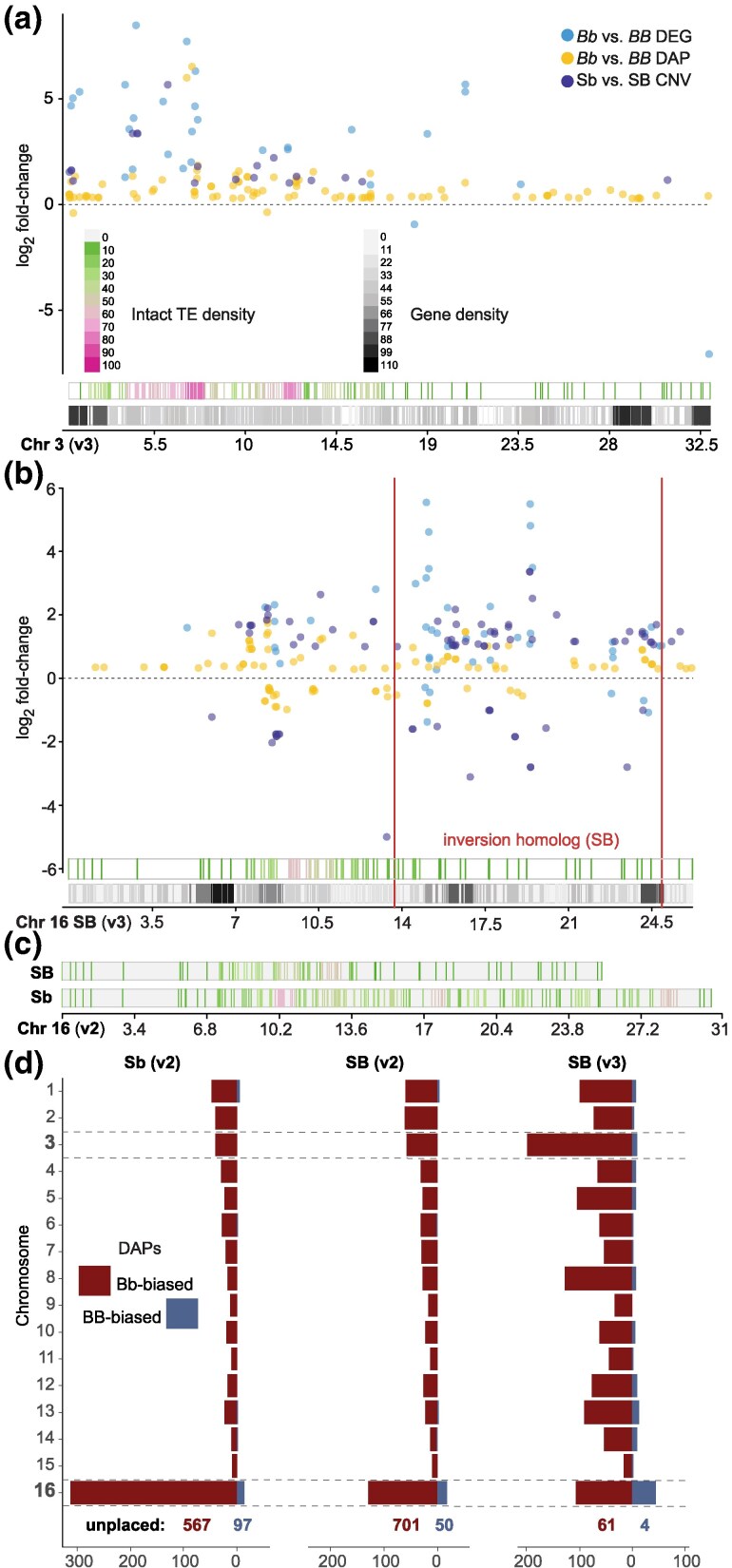
Intact TE density and the genomic distribution of social chromosome regulatory effects. a) Chromosome (Chr) 3 location of DEGs, DAPs, and CNVs with respect to genes and intact TEs. b) *SB* Chr 16 location of DEGs, DAPs, and CNVs with respect to genes and intact TEs. Red lines demark the external bounds of supergene inversion breakpoints in *Sb*. c) Intact TE density for *SB* and *Sb* long-read social chromosome assemblies illustrates rapid evolutionary gain of TEs in *Sb* relative to *SB*. d) Distribution of DAPs by chromosome on comparable *Sb* and *SB* long-read genome assemblies (v2) (Yan et al. 2020) and an improved *SB* assembly (v3) (Helleu et al. 2022) show trans-regulatory effects of the supergene and prevalence of DAPs on the social chromosome (Chr 16) and unplaced scaffolds.

In line with the positive correlation between TE density and DAPs, a visual examination of chromosome 3 (enriched for orange module genes) illustrates an apparent hotspot of intact TEs in a region with many DEGs, DAPs, and CNVs (Fig. 3a). The centromere-proximal region of the social chromosome also exhibits moderate to high intact TE density and harbors many DEGs, DAPs, and CNVs (Fig. 3b, left of red lines). By contrast, the region of the *SB* supergene haplotype that is homologous to the *Sb* chromosomal inversion region coincides with a paucity of TEs (Fig. 3b, between red lines). We used a reference genome that includes the *SB* haplotype of the social chromosome for most of our analyses because the currently available *Sb* genome assembly is less complete and does not have equivalent gene annotations (however, we do consider effects of alternative genome assemblies on our results below). Following its origin, the *Sb* supergene haplotype did accumulate many intact TEs (Stolle et al. 2019; Yan et al. 2020), as confirmed by a comparison of methodologically equivalent long-read genome assemblies for *SB* and *Sb* social chromosome haplotypes (which we refer to as v2 (Yan et al. 2020); Fig. 3b-c). Prior optical mapping and *k*-mer analyses suggest that the full extent of *Sb* expansion represents an increase in length of over 30% relative to *SB* (Stolle et al. 2019). Overall, these results provide preliminary evidence that TE density may be important to the patterns of differential gene expression and chromatin accessibility observed by supergene genotype, including but not limited to TEs within the expanded *Sb* supergene haplotype.

Across the genome, genes with evidence of *Sb*-linked CNVs (Fontana et al. 2020) also exhibited a significantly increased overlap with intact TEs as compared to non-CNVs (supplementary fig. S1, Supplementary Material online), with 19.47% of CNVs overlapping at least 1 intact TE as compared to 6.41% of non-CNVs overlapping at least 1 intact TE (Fisher's exact test, odds ratio = 3.6, *P* = 1.3e-12). This suggests that TEs are likely to have played a direct role in the translocation and duplication of host genes in the social chromosome, consistent with the importance of TEs to the origins of CNVs more generally (Cerbin and Jiang 2018) and observations of TEs flanking some of the CNVs (Fontana et al. 2020) and one of the large inversions in the fire ant supergene (Yan et al. 2020).

Structural differentiation of heteromorphic chromosomes complicates analyses of gene regulation because cells of diploid organisms include 2 chromosome variants whereas most analysis pipelines involve mapping to a single reference genome to identify differential activity. To consider such effects on our analyses and interpretation, we assessed how the chromosomal distribution of differentially accessible chromatin peaks by supergene genotype might be influenced by mapping our ATAC-seq data to an *Sb* versus *SB* reference genome assembly and *SB* reference assemblies of variable completeness. Mapping to an *Sb* reference assembly resulted in over twice as many DAPs being assigned to chromosome 16 as compared to the comparable *SB* assembly (Fig. 3d; supplementary fig. S2 and table S12, Supplementary Material online). This suggests some DAPs that were on unplaced scaffolds or mapped to other chromosomes in an *SB* reference genome may actually be *Sb*-linked CNVs with new duplicates present in the supergene (at present, we do not have the chromosome level assembly and gene models for *Sb* needed to fully disentangle this issue directly). When we analyze DAPs mapped to the *Sb* (v2) genome, which are not dependent on gene annotations, the signal of the hotspot of intact TEs and DAPs in chromosome 3 (Fig. 3a) remains intact (supplementary figs. S2 and S3, Supplementary Material online). Moreover, the most recent assembly of *SB* shows great reduction in unmapped content (v3, available for only *SB*) (Helleu et al. 2022) as well as increases in *Sb*-biased DAPs in many chromosomes other than the social chromosome (Figs. 1f and 3d) relative to the less complete *SB* assembly (v2) (Yan et al. 2020), consistent with our hypothesis that regions that harbor intact TEs (which are difficult to resolve in assemblies) are frequent targets of trans-regulation by the supergene. Regardless of genome build used, DAPs were retained on every chromosome and universally exhibited an enrichment of increased accessibility in *Sb* (Fig. 3d; supplementary fig. S2, Supplementary Material online; 75.87%, 89.01, and 89.75% of all DAPs mapped to chromosomes or scaffolds other than chromosome 16 in builds *Sb* v2, *SB* v2, and *SB* v3, respectively).

The variation in DAP distributions we observed across chromosomes and unplaced scaffolds between reference genome builds are consistent with an evolutionary history of *Sb* that includes some combination of translocation and copy number expansion. Nevertheless, many DEGs and DAPs that exhibit a particularly strong bias toward higher expression and chromatin accessibility in individuals carrying the *Sb* variant of the social chromosome do not exhibit evidence of supergene-linked copy number variation (Fig. 2b) (Fontana et al. 2020). Overall, our analyses of gene expression, chromatin accessibility, CNVs, and intact TEs suggest that the *Sb* supergene exhibits trans-regulatory effects that are influenced by a combination of copy number variation, gene regulatory evolution, and, potentially, effects of TE density on trans-regulatory activation of host genes.

### TFs May Be Drivers of Global Effects of the Social Chromosome

Based on the widespread effects of social chromosome genotype on chromatin accessibility and gene activity in the *S. invicta* genome (Fig. 1), we hypothesized the regulation of some TFs may be influenced by social chromosome evolution. Indeed, examination of the orange gene co-expression module revealed that 9 of the 44 genes in the module are either orthologs of TFs or harbor DNA binding domains (Fig. 2b). Three distinct (non-CNV) uncharacterized proteins with Myb_SANT_like DNA binding domains are included in this total (described in relation to HARBI1 genes above), as well as a zinc finger BED domain-containing protein-encoding gene and several uncharacterized genes with zinc finger protein domains (Fig. 2b). Despite the constitutive effects of the supergene on expression in the orange module, none of the putative TFs in the module exhibit evidence of CNV between SB and Sb (Fig. 2b). These putative TFs nevertheless stand to play a particularly important role in trans-regulatory effects of the social chromosome.

We further identified 8 putative TFs with evidence of *Sb*-linked CNV (Fontana et al. 2020) that lack constitutive variation in expression by supergene genotype (Table 2). Through context-specific regulation, these CNVs may convey direct trans-regulatory effects of the social chromosome, including in early developmental stages and specific tissues that have yet to be examined. Among the TFs exhibiting CNV between *Sb* and *SB*, 4 were also found to exhibit differential expression in 1 of 2 examined tissues of adult workers or queens between *SB/SB* and *SB/Sb* genotypes and 4 are near DAPs between *SB/Sb* and *SB/SB* worker brains (Table 2). Among orthologs of these genes, perturbation of both *cnc* (Deng and Kerppola 2013) and *btsz* (Serano and Rubin 2003) has been shown to influence body size at pupation in *Drosophila*. *Solenopsis invicta* orthologs of *cnc* and *btsz* thus represent excellent candidate factors for mediating effects of supergene genotype on worker size and queen determination (Buechel et al. 2014).

**Table 2:** TFs with social chromosome-linked copy number variation

Sinv ID	Fly ID	NCBI description	CNV log_2_ b/B^a^	DEG log_2_ Bb/BB^b^	DAP	Chr	TF annotation^c^
LOC105193811	CG7786	Cell death specification protein 2	1.06	1.65 (W_ga_)		16	GO:0003700
LOC105193215	cnc	Segmentation protein cap'n'collar	1.16			16	GO:0003700
LOC105197366	trr	Histone-lysine N-methyltransferase 2C	−1.60		Bb > BB	16	Fly ortholog
LOC105196676	Shab	Potassium voltage-gated channel protein Shab	2.17		Bb > BB; BB > Bb	4	Fly ortholog
LOC105196046	btsz	Serine-rich adhesin for platelets	1.75		Bb > BB	12	Fly ortholog
LOC113005811		Uncharacterized	1.08	3.55 (W_ga_)	Bb > BB	13	HTH
LOC105199862	CG30428	Uncharacterized	1.91	−5.11 (Q_br_)		11	Myb_SANT_like
LOC113004138		Putative uncharacterized protein DDB_G0282133	1.35	1.56 (W_br_)		2	BTB_POZ

^a^ Fontana et al. (2020).

^b^W_ga_, worker gaster; Q_br_, alate gyne (queen) brain; W_br_, worker brain.

^c^Evidence of potential TF activity (lowest number in list used as priority): (i) annotation with GO:0003700 “DNA-binding transcription factor activity”, (ii) orthology with *D. melanogaster* protein (“Fly ortholog”), or (iii) InterPro or Panther DNA-binding domain description.

### Copy Number Variation and Enhancer Evolution Act in Concert to Shape the Expression of a Supergene

To assess whether the social chromosome supergene and associated genotypic differentiation between *SB* and *Sb* alters transcriptional enhancer activity, we employed STARR-seq, a high-throughput reporter assay that quantifies the enhancer activity of randomly sheared genomic fragments (Arnold et al. 2013, 2014; Jones et al. 2024). Using *Drosophila melanogaster* embryonic S2 cells, we identified 23,616 regions of the *S. invicta* genome with significant enhancer activity in either genotype (pools of either *SB* or *Sb* individuals, see Methods) (Jones et al. 2024), 11,670 of which were active in both genotypes (supplementary table S13, Supplementary Material online). Enhancers were located primarily within introns (51.8%), promoter regions (13.9%), and transcription start sites (TSSs, 200 bp flanking regions; 11.8%) of 5,293 genes (supplementary fig. S4, Supplementary Material online). Supportive of the potential regulatory function of these regions, STARR-seq peaks were significantly more likely to overlap accessible chromatin peaks according to ATAC-seq than expected by chance (supplementary fig. S5, Supplementary Material online; permutations, 1.8-fold enrichment, *P* < 0.005).

Unlike our finding of consistently more accessible chromatin in *Sb*-containing individuals, we did not see the same bias for enhancer activity when comparing SB and Sb DNA pools (i.e. the numbers of regions with biased activity toward *Sb* or *SB* pools were similar; supplementary fig. S6 and table S13, Supplementary Material online). This apparent discordance between regulatory activity of *Sb* inferred from ATAC-seq and STARR-seq suggests that while underlying sequence variation between genotypes influences enhancer activity, it is critical to also consider epigenetic context to understand how sequence variation leads to different phenotypes. For example, overlapping our enhancer set with DAPs suggests that approximately 12% of differentially accessible chromatin regions are enhancers (174/1454 DAPs overlap enhancer regions identified by STARR-seq). These peaks may be especially informative for understanding how supergene-linked variation influences gene regulation. Genes proximal to all enhancers with *SB*-biased activity (*n* = 3,378) were enriched for 81 GO terms, including terms related to signal transduction, cell adhesion, metabolism, axon guidance, and synapse organization (supplementary table S14 and fig. S7, Supplementary Material online), while genes proximal to all *Sb*-biased enhancers (*n* = 4,079) were enriched for 61 GO terms related to transcriptional and protein biosynthetic processes, as well as terms related to neuronal processes (supplementary table S15 and fig. S8, Supplementary Material online).

Importantly, while sequence-based differences in enhancer activity are revealed by STARR-seq, the availability of upstream regulatory proteins and chromatin modifications will influence how this enhancer activity is realized in vivo. In addition, the pools of DNA used for STARR-seq included random genetic variation unrelated to social forms. Thus, we leveraged existing population genetics data (Wang et al. 2013; Martinez-Ruiz et al. 2020) (supplementary table S16, Supplementary Material online) to identify the location of variable loci between *SB* and *Sb* and tested for associations of these alleles with enhancer variation. First, we identified 283,725 loci with variation in the published data sets, which included US (Wang et al. 2013) (*n* = 7 SB and Sb) and Argentine (Martinez-Ruiz et al. 2020) (*n* = 13 SB and Sb) populations of *S. invicta*. Of the identified variants, nearly 20% (55116/283725) were located within enhancer peaks. While most of these variants were not fixed between *SB* and *Sb* genotypes, we identified 797 loci with differences in allele frequencies > 80% between genotypes, 160 of which were divergent in both populations. Of those sites, 94 (of 797) were located within enhancer regions, providing a list of candidate loci that may influence regulatory activity. To assess the putative function of these loci, we tested for predictive effects of these SNPs on TF binding using the FIMO tool of MEME suite and the JASPAR 2024 set of core insect TF motifs (Rauluseviciute et al. 2024). Considering a region centered on the SNP with 50 bp flanks, 49 of the 94 candidate regions had significant effects on at least 1 TF binding motif (supplementary table S17, Supplementary Material online). Motifs with the greatest numbers of predicted effects included those for Mothers against dpp (Mad), Clamp, Odd paired (Opa), and Dorsal (Dl), each of which had changes in predicted binding at 4 loci (supplementary table S17, Supplementary Material online). These TFs have numerous known roles through development (Jenkins et al. 2022), but Mad, Clamp, and Opa have caste-biased occupancy in honey bees (Lowe et al. 2022) and Clamp has also been associated with the major worker phenotype in ants (Glastad et al. 2020), suggesting these TFs may be frequently coopted for social-related phenotypes during evolution. It is notable in this context that *SB/Sb* larvae are more likely than *SB/SB* larvae to develop into large (major) workers and gynes (Buechel et al. 2014).

**Fig. 4.**
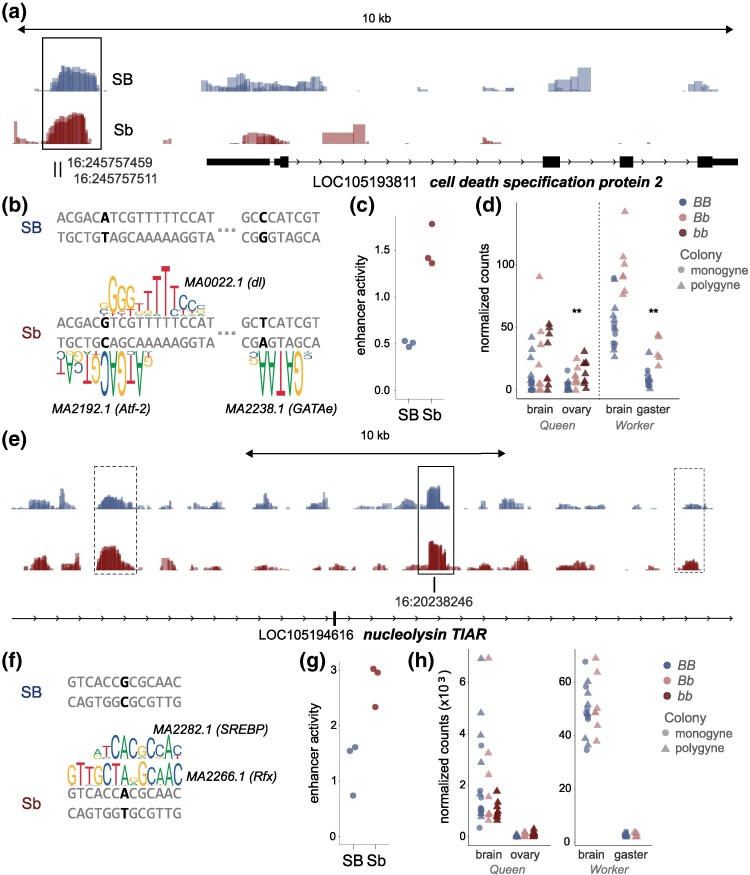
Differential enhancer activity coinciding with substitutions that affect predicted TF binding. a) Enhancer activity (displayed as bigwig track of log2 normalized ratios between mRNA and input DNA per replicate) according to STARR-seq, with increased enhancer activity in *Sb* relative to *SB* for an enhancer (within rectangle) in the upstream region of the TF LOC105193811 *cell death specification protein 2* and b) effects of 2 substitutions between *Sb* and *SB* on predicted TF binding within the enhancer. c) Increased enhancer activity in *Sb* based on STARR-seq mirrors the increase in predicted binding of TFs. d) Gene expression of *Sb*-carrying individuals is elevated in alate gyne (queen) ovaries and worker gasters, based on previously published data (Arsenault et al. 2020, 2023). e) Enhancer activity according to STARR-seq, wherein 3 intronic enhancers of LOC105194616 *nucleolysin TIAR* show *Sb*-biased activity, including an enhancer (solid rectangle) with a substitution in *Sb* relative to *SB*. f) Effects of a substitution between *Sb* and *SB* on predicted TF binding within the enhancer. g) Increased activity of enhancer in *Sb* based on STARR-seq. h) Gene expression is not significantly different by genotype in queens or workers for this gene.

Of the predicted motif effects of divergence between *Sb* and *SB*, 17 were consistent with differences in enhancer activity between *SB* and *Sb* sequences based on STARR-seq, including 6 SNPs with fixed differences across both populations (United States and Argentina) and significant correlations between allele frequencies at these loci and enhancer activity. These 6 variants are located within the supergene region in enhancers upstream or intronic to 4 genes, including *LOC105195710*, *cytoplasmic polyadenylation element-binding protein 2*, *cell death specification protein 2*, and *nucleolysin TIAR* (Fig. 4). Future work should investigate the functional effects of these variants on TF binding as well as gene expression of putatively regulated genes.

Our integrative analysis of copy number variation, enhancer activity, and sequence substitutions between *SB* and *Sb* provides insight into how copy number variation may interact with enhancer evolution to fine tune gene expression. Among the putative TFs with copy number variation and orthologs of known function, we observed a significant differentially active enhancer (DAE), with higher activity in *Sb* than in *SB* in the upstream promoter region of LOC105193811 *cell death specification protein 2* (Fig. 4a; log2(*b/B*) 1.03, q-val = 0.0003), a TF encoding gene that also has an extra copy in *Sb* (Fontana et al. 2020). Within this DAE, we observed 2 substitutions between *SB* and *Sb*. Both variants affect the predicted binding affinity of TF binding motifs according to FIMO (supplementary table S17, Supplementary Material online), all of which lead to increased predicted binding of the *Sb* versus *SB* sequence (Fig. 4b), providing a putative mechanism for the increased enhancer activity (Fig. 4c) of the *Sb* sequence. These data suggest cis-regulatory evolution has acted to increase expression of *cell death specification protein 2* in *Sb*-carrying individuals. In this case, cis-regulatory evolution appears to have acted in concert with structural evolution to increase expression of a putative TF. As further evidence of regulatory significance, we note that *cell death specification protein 2* is differentially expressed between *SB/Sb* and *SB/SB* in worker gasters (log2 ratio 1.65, FDR = 0.001) and in alternative homozygotes (*Sb/Sb* vs. *SB/SB*) in queen ovaries (log2 ratio 3.88, FDR = 0.009) (Arsenault et al. 2020) (Fig. 4d).

Our analysis also revealed a potential example of evolutionary gene dosage compensation in the context of copy number variation for LOC105194616 *nucleolysin TIAR*, which encodes a protein that likely binds RNA and targets lysosomes to help induce apoptosis (Kawakami et al. 1992). This gene exhibits 2 copies in the ancestral *SB* (also *LOC105196096*) but has lost a copy in *Sb* (supplementary table S7, Supplementary Material online) (Fontana et al. 2020) and is not differentially expressed between *SB/Sb* and *SB/SB* samples. We observed 3 intronic enhancers of *LOC105194616*, one of which contained a fixed variant and showed higher activity in *Sb* compared with *SB* (Fig. 4e; log2(b/B) 1.48, q-val = 0.0003). The substitution between *SB* and *Sb* in this DAE affects the predicted binding affinity of 2 different TFs according to FIMO, each of which has increased binding of the *Sb* versus *SB* sequence (Fig. 4f, supplementary table S17, Supplementary Material online), providing a putative mechanism for the increased enhancer activity of the *Sb* sequence (Fig. 4g). In this case, cis-regulatory evolution appears to have acted in concert with structural evolution to maintain the correct dosage of *nucleolysin TIAR* (i.e. this gene is not differentially expressed between genotypes despite copy number variation; Fig. 4h).

## Conclusion

The fire ant supergene is evolutionarily young but structurally diverse, providing an ideal model to examine how regulatory sequence divergence, CNVs, and TEs interact to influence genome regulation. Our investigation revealed that the derived supergene haplotype is associated with widespread increases in chromatin accessibility in genomic regions containing intact TEs. Thus, a relationship between chromatin accessibility and TF binding sites associated with transposon activation could potentially contribute to the observed prevalence of *Sb*-activating effects on gene expression. Such a scenario would also be consistent with our finding of *Sb* trans-upregulation of genes domesticated from an ancestral transposon. Importantly, our results highlight that multiple orthologs of functionally annotated *Drosophila* TFs exhibit social chromosome-linked CNVs in *S. invicta*. Enhancer activity differences by supergene haplotype revealed that regulatory element sequence evolution can amplify or mitigate effects of such CNVs. Overall, this research highlights that CNVs and regulatory sequences evolve in concert to shape the expression of supergene encoded factors with trans-regulatory links to the remainder of the genome.

## Methods

### Gene Expression

#### Data Sources

We compiled RNA-seq data from 122 libraries (supplementary table S1, Supplementary Material online), detailed as follows: Chandra et al. (2018) contributed 20 brain libraries split among workers (4 *SB/SB*; 6 *SB/Sb*), gynes (2 *SB/SB*; 5 *SB/Sb*), and reproductive queens (3 *SB/Sb*) from lab-reared colonies originally collected in Gainesville, Florida. Arsenault et al. (2020) provided 63 brain and ovary libraries from 32 individual gynes (referred to as queens in main text) collected while embarking on spring mating flights around Athens, Georgia, United States, with 8 each of monogyne brain and ovary (all *SB/SB*) and 8 polygyne trios in both tissues (*SB/SB*, *SB/Sb*, *Sb/Sb*). Arsenault et al. (2023) added 39 libraries from individual monogyne workers (7 brain, 6 gaster; all *SB/SB*) and polygyne workers (7 brain and 6 gaster libraries each of *SB/SB* and *SB/Sb*) collected from age and size matched 2-week old adult workers from lab-reared colonies originally collected around Gainesville, Florida, United States. For further RNA-seq data generation details, see the source publications.

#### Quality Control and Read Alignment

We trimmed reads and performed quality control using Trim Galore! v0.6.7. We then used STAR v2.7.10b (Dobin et al. 2013) to align reads using the 2-pass alignment procedure to the UNIL 3.0 genome assembly and associated annotation generated from an *SB* haploid male (GCF_016802725.1) (Helleu et al. 2022). After alignment, single-end worker libraries (Chandra et al. 2018) had 6 to 15 million, single-end gyne libraries (Arsenault et al. 2020) had 11 to 32 million uniquely mapped reads, and paired-end worker libraries (Arsenault et al. 2023) had 32 to 163 million. We loaded quantified, gene-level read counts found using featureCounts (Liao et al. 2014) into R Statistical Software (R_Core_Team 2024; version 4.3.1) for subsequent analyses.

#### Differential Expression Analysis

We used DESeq2 (Love et al. 2014) to perform separate differential expression analyses for gyne data from Arsenault et al. (2020) and worker data from Arsenault et al. (2023) (supplementary table S1, Supplementary Material online). Pairwise DEGs of primary interest were contrasts between *SB/Sb* and *SB/SB* samples of the same tissue from the same caste and experiment (to control for batch effects), all collected from a polygyne colony social environment (supplementary table S2, Supplementary Material online). To visualize expression variance and relationships between libraries, HCA was performed with the R package pheatmap (Kolde and Kolde 2018) using the “ward D2” clustering method. All DEGs were called using a 5% false discovery rate threshold (FDR < 0.05). All pairwise comparisons of gene expression are provided in supplementary table S2, Supplementary Material online.

#### Weighted Gene Co-expression Network Analysis

Normalized counts from all 122 libraries were converted to an expression matrix for WGCNA (v.1.72-5) (Langfelder and Horvath 2008). We applied a low expression filter to remove genes that had a normalized count value less than 10 for more than 90% (109/122) of the libraries. We then performed a variance stabilizing transformation using varianceStabilizingTransformation from DESeq2 on the filtered raw expression data. To visualize expression variance and relationships between all libraries, HCA was performed with the R package pheatmap (Kolde and Kolde 2018) using the “ward D2” clustering method. We also performed a PCA using all samples (supplementary fig. S9, Supplementary Material online).

Adjacency matrices were raised to a soft power threshold of 24. This was empirically determined based on a measure of *R*^2^ scale-free topology model fit that maximized and plateaued over 0.9. The soft-power thresholded adjacency matrices were converted into a topological overlap matrix (TOM) and a topological dissimilarity matrix (1-TOM). We then performed agglomerative hierarchical clustering using the average linking method on the TOM dissimilarity matrix. Gene modules were defined from the resulting clustering tree (supplementary fig. S10, Supplementary Material online), and branches were cut using the hybrid dynamic tree cutting function: the module detection sensitivity (deepSplit) was set to 2, minimum module size 20, and the cut height for module merging set to 0.15 (modules whose eigengenes were correlated above 0.85 were merged). This yielded 26 consensus modules which were each assigned a color label (supplementary table S8, Supplementary Material online). For each gene module, a summary measure (module eigengene) was computed as the first principal component of the module expression profiles.

### Chromatin Accessibility

#### Worker Brain Cell Suspension for ATAC-seq

Individual adult workers of unknown age were sampled from a lab-reared colony initially collected around Athens, Georgia, United States. Brains of *S. invicta* workers were dissected under an Olympus SZ61 microscope and stored individually in 1.5 mL microcentrifuge tubes containing 50 µL Hank's Balanced Salt Solution (HBSS) (H6648-500ML, Sigma). Tubes were kept on ice to preserve the cells. Nine hundred and fifty microliters of a solution of Papain (LK003178, Worthington Biochemical) mixed with 5 mL HBSS was added to each tube, and the tubes were incubated in a thermal mixer (13687720, Thermo Scientific) for 15 min at 25 °C, with 900 RPM rotation/mixing. After incubation, the Papain-HBSS buffer was removed and brains were resuspended in 200 µL phosphate-buffered saline (PBS) (806544-500ML, Sigma) + protease inhibitor complex (PIC) (Roche 4693132001). Each brain was then aspirated in 100 µL PBS-PIC, transferred to a glass dounce, and gently homogenized with 2 pestles. The homogenate was transferred to a new 1.5 µL microcentrifuge tube; the dounce and pestles were rinsed in the remaining 100 µL PBS-PIC, and the rinsate was added to the new tube, to increase recovery.

#### ATAC-seq Transposition Reaction

Cells were pelleted at 500 RCF (0.5 × 1000) at 4 °C for 5 min, after which PBS-PIC supernatant was removed, and 50 µL of cold ATAC-Resuspension Buffer (RSB) (500 µL 1 M Tris-HCL [15567-027, Invitrogen], 100 µL 5 M NaCl [AM9759, Ambion/Thermo], 150 µL 1 M MgCl2[AM9530G, Ambion/Thermo], 49.25 mL sterile H20) containing 0.1% NP40 substitute (2127-50, BioVision), 0.1% Tween-20 (11332465001, Sigma/Roche), and 0.01% Digitonin (G9441, Promega) was added to lyse cells. The RSB containing detergents was gently pipetted up and down 3 times, and the mixture was incubated on ice for 3 min. To inhibit lysis, 1 mL of cold ATAC-RSB containing only 0.1% Tween-20 was then added, and tubes were inverted 3 times. Nuclei were pelleted at 500 RCF for 10 min at 4 °C.

All supernatant was aspirated, taking care to avoid the cell pellets, and the pellets were resuspended in 50 µL of transposition mixture (25 µL 2× TD buffer, 2.5 µL transposase (100 nM final), 16.5 µL PBS, 0.5 µL 1% Digitonin, 0.5 µL 10% Tween-20, 5 µL H20) by pipetting up and down 6 times. The reaction was incubated at 37 °C for 30 min in the thermal mixer described above with 1,000 RPM mixing. Reactions were cleaned using a DNA Clean and Concentrator-5 Kit (D4014, Zymo), using 250 µL DNA Binding Buffer. DNA was eluted in 25 µL elution buffer and stored at −20 °C until amplification.

#### Amplification of Transposed Fragments and Sequencing

Transposed fragments were amplified using the following PCR recipe: 25 µL 2× NEBNext® High-Fidelity 2× Master Mix (M0541L, NEB), 2.5 µL of 25 µM i5 primer, 2.5 µL of 25 µM i7 primer, and 20 µL transposed/cleaned-up reaction. Different i7 and i5 primer combinations were used for each reaction. The thermocycler conditions are as follows: 72 °C for 5 min and 98 °C for 30 s, followed by 9 cycles of 98 °C for 10 s, 63 °C for 30 s, and 72 °C for 1 min, and final hold at 4 °C. PCR products were purified with a DNA Clean and Concentrator-5 Kit (D4014, Zymo) using 250 µL DNA Binding Buffer and eluted in 25 µL sterile H20.

Fragment analysis on a bioanalyzer supported nucleosomal banding patterns for libraries selected for sequencing (supplementary fig. S11, Supplementary Material online). ATAC-seq libraries were pooled and sequenced on an Illumina NextSeq 2000 with 50 bp paired-end reads (supplementary table S3, Supplementary Material online). After filtering, an average of 121 million read pairs remained for each library (range: 108 to 142 million), 97% of which were mapped in proper pairs to the assembly with duplication rates of 24% to 27%. Additional information on sequencing coverage and alignment is in supplementary table S3, Supplementary Material online. Mean genomic coverage across samples ranged from 20.0 to 26.9 reads per bp (supplementary fig. S12, Supplementary Material online).

#### ATAC-seq Analyses

FastQC (Andrews 2010) was used to examine data quality, and raw reads were trimmed to remove low-quality bases and sequencing adapters with fastp v0.20.1 (–cut_right, –cut_right_window_size 4, –cut_right_mean_quality 15) (Chen et al. 2018). Trimmed reads were mapped to the *S. invicta* genome assembly UNIL_Sinv_3.0 with BWA v0.7.17 (bwa mem –M) (Li 2013) and sorted by read name using samtools v1.3 (samtools sort –*n*) (Danecek et al. 2021). Genrich v0.6.1 (https://github.com/jsh58/Genrich) was used to call peaks on each sample group (*BB* and *Bb*, including all replicates of each type in the respective peak calling runs) in ATAC-seq mode with removal of PCR duplicates (Genrich –j –y –r). Genrich analyzes multiple replicates collectively and combines *P*-values from those individual replicates to obtain q-values and generate group-level peaks, which we used to obtain *SB/SB* and *SB/Sb* peak sets. Merging Genrich peaks from both sample groups resulted in 33,619 total peaks, with an average of 31.4% of reads mapping to this peakset (Fraction of Reads in Peaks, or FRiP, ranged from 0.3015 to 0.3307; supplementary table S3, Supplementary Material online). These peaks were assigned to gene features using the publicly available RefSeq annotation GCF_016802725.1_UNIL_Sinv_ 3.0_genomic.gff, with TSS flanks defined as the TSS ± 200 bp, promoters defined as 0 to 5 kb upstream of gene start, upstream regions defined as 0 to 10 kb upstream of gene start, and downstream regions defined as 0 to 10 kb downstream of gene start. Exons and introns were extracted from the RefSeq annotation. Peaks that did not overlap any of these features were annotated as intergenic, and peaks that overlapped multiple regions were assigned in the following priority: TSS flank, promoter, intron, upstream, downstream, exon, and intergenic. The function overlapPermTest in the R package regioneR (Gel et al. 2016) was used to assess whether the overlap of peaks with particular features was more than expected by chance. ATAC-seq peaks were enriched for the following features: TSS flanks, promoters, upstream regions, downstream regions, and exons (supplementary fig. S13, Supplementary Material online). In contrast, ATAC-seq peaks overlapped introns and intergenic regions less often than expected by chance (supplementary fig. S13, Supplementary Material online).

DAPs (FDR < 0.1; supplementary table S4 and fig. S14, Supplementary Material online) were identified using DiffBind v3.12.0 (https://bioconductor.org/packages/release/bioc/html/DiffBind.html) with default DESeq2 parameters using deduplicated BAM files (produced with picardtools v2.22.1 [http://broadinstitute.github.io/picard] following coordinate-sorting of BAMs by samtools) and Genrich merged peaks as input regions of interest. Peaks were annotated as detailed above with both with the nearest priority-assigned gene and to all genes within 10 kb of either end of the peak.

### Enhancer Activity

#### Generation of STARR-seq Plasmid Libraries

Genomic DNA was isolated from individual male progeny of a lab-reared isolated *S. invicta* polygyne queen and a polygyne colony containing multiple queens, both collected around Athens, Georgia, United States, using a Zymo Quick-DNA Tissue/Insect Microprep Kit (Zymo Research cat. no. D6015). Extracted DNA from each individual male was genotyped twice at *Gp-9* to determine whether they were hemizygous or homozygous at the *Gp-9* locus, resulting in 9 males with *SB* and 10 males with *Sb*. These *SB* and *Sb* samples were pooled to obtain sufficient material to generate *SB*- and *Sb*-specific STARR-seq plasmid libraries. Additional details on individuals used for each genotype are in supplementary table S18, Supplementary Material online. A pool of 5 µg from each genotype was sonicated in a microTUBE with AFA fiber (cat. no. 520045) using a Covaris LE220 and the following parameters: 45 s sonication time, 450 W peak incident power, 15% duty factor, and 200 cycles per burst. Sheared DNA was run on a 1% agarose gel, and 400 to 750 bp fragments were size-selected via excision under blue light. Size-selected DNA was purified using a Gel DNA Recovery Kit (Zymo Research cat. no. D4008), followed by an additional purification with the QIAquick PCR Purification Kit (Qiagen cat. no. 28104). Size-selected DNA was quantified using a Qubit dsDNA HS Assay Kit (Invitrogen cat. no. Q32854) on a Qubit4 Fluorometer.

Illumina-compatible adapters were ligated to size-selected genomic DNA using the NEBNext Ultra II End Repair Module (NEB E7546L) with 1 µg fragmented DNA per genotype. Adapter-ligated DNA libraries were then cleaned with a 1.8× volume ratio of AMPure XP Reagent beads (Beckman Coulter cat. no. A63881) to sample following manufacturer protocols for PCR purification, then cleaned a second time with 0.8× bead to sample volume ratio. Adapter-ligated DNA libraries were then amplified in 5 separate reactions for 5 cycles (PCR conditions: initial denaturation of 98C for 45 s, then 5 cycles of (i) denaturation: 98 °C for 15 s, (ii) annealing: 65 °C for 30 s, (iii) elongation: 72 °C for 45 s, followed by a final elongation at 72 °C for 60 s) using in_fusion_F (TAGAGCATGCACCGGACACTCTTTCCCTACACGACGCTCTTCCGATCT) and in_fusion_R (GGCCGAATTCGTCGAGTGACTGGAGTTCAGACGTGTGCTCTTCCGATCT) primers at 10 µM and 2× KAPA HiFi HotStart Ready Mix (Roche cat. no. KK2601). PCR reactions were pooled for each genotype and cleaned with 0.8× bead to sample volume ratio with AMPure XP Reagent beads (Beckman Coulter cat. no. A63881) followed by an additional purification using the QIAquick PCR Purification Kit (Qiagen cat. no. 28104). Libraries were quantified using a Qubit dsDNA HS Assay Kit (Invitrogen cat. no. Q32854) on a Qubit4 Fluorometer, and average sizes of each adapter-ligated, amplified library were determined with Agilent High Sensitivity DNA reagents on an Agilent 4200 TapeStation (Agilent cat. nos. 5067-5592, 5067-5593, 5067-5594).

pSTARR-seq_fly was a gift from Alexander Stark (Addgene plasmid #71499; http://n2t.net/addgene:71499; RRID:Addgene_71499) (Arnold et al. 2013). The pSTARR-seq_fly reporter vector was digested with AgeI-HF (NEB cat. no. R3552S) and SalI-HF (NEB cat. no. R3138S) restriction enzymes with 250 units of each and 25 µg vector per reaction, incubated at 37 °C for 2 h followed by a 20 min heat inactivation at 65 °C. Digested products were run on a 1% agarose gel, and linearized vector was selected via excision under blue light and purified using a Gel DNA Recovery Kit (Zymo Research cat. no. D4008). Eluates from gel extraction were purified using a 1.8× bead cleanup with AMPure XP beads (Beckman Coulter cat. no. A63881). Adapter-ligated DNA libraries were cloned into purified pSTARR-seq_fly using a 2:1 molar ratio of insert (size determined via TapeStation) to plasmid (∼4125 bp). Two cloning reactions were conducted for each genotype using 1 µg digested plasmid, the appropriate amount of PCR amplified, adapter-ligated DNA library to have a 2× molar excess insert to plasmid, and 10 µL 5× In-Fusion HD Enzyme Premix (Takara cat. no. 638910) in a total volume of 50 µL. Reactions were incubated for 15 min at 50 °C, then 200 µL EB was added. Next, 25 µL 3 M NaAc (pH 5.2) and 2 µL Pellet Paint Co-precipitant (Millipore cat. no. 69049) were added to each reaction, vortexing between each addition. Finally, 750 µL ice-cold 100% EtOH was added, and samples were vortexed and then incubated at −20 °C for 16 h. Samples were centrifuged for 15 min at full speed and 4 °C, vortexed, and centrifuged again for 15 min, and then, supernatant was carefully aspirated. Cloned DNA pellets were washed 3 times with 750 µL ice-cold 70% EtOH, mixing each time by inversion. Cloned DNA pellets were again centrifuged for 15 min at full speed and 4 °C, supernatant aspirated, then pellets dried for 30 s at 37 °C, and then further at room temperature until dry. Each pellet was resuspended in 12.5 µL EB and incubated for 3 h at −80 °C prior to transfer to −20 °C for storage until transformation.

Cloned DNA reactions were transformed into electrocompetent MegaX DH10B cells (Thermo Fisher cat. no. C640003) using 150 µL cells for each clone (2 clones per genotype) split across 2 Gene Pulser Electroporation Cuvettes (0.1 cm gap, Bio-Rad cat. no. 1652089) and the Bio-Rad Gene Pulser Xcell system with the following electroporation conditions: 2 kV, 25 µF, and 200 ohms. Immediately after electroporation, 500 µL of prewarmed recovery medium was added and cells were transferred to round bottom tubes with an additional 4 mL warm recovery media. Transformed bacteria were incubated for 1 h at 37 °C and 225 rpm, and then, each transformation reaction was added to 300 mL warm LB + ampicillin (100 µg/mL) in 2 L flasks and incubated for 12 h while shaking at 200 rpm at 37 °C. Cells were harvested via centrifugation and plasmids were purified using the ZymoPure II Plasmid Maxiprep Kit (Zymo Research cat. no. D4203) with a maximum of 75 mL culture per column and eluted in water heated to 50 °C prior to elution. Plasmids were pooled within each clone and then across clones from the same genotype, resulting in 1 clone library per genotype (i.e. *SB* library and *Sb* library).

#### Transfection of Drosophila Cells with STARR-seq Plasmid Libraries

Three replicate flasks were seeded and transfected for each genotype (*SB* or *Sb*). *Drosophila* S2 cells (S2-DRSC; DGRC Stock 181; https://dgrc.bio.indiana.edu//stock/181; RRID:CVCL_Z992) (Schneider 1972) were cultured in M3 media (Sigma-Aldrich cat. no. S8398) supplemented with BactoPeptone (BD Biosciences #211677), yeast extract (Sigma-Aldrich Y1000), 10% heat-inactivated fetal bovine serum (Gibco cat. no. 10437-010), and 1% penicillin/streptomycin solution (Thermo Fisher cat. no. 15140122) at 25 °C using standard cell culturing protocols. Twenty-four hours prior to transfection, cells were split, washed, counted, and seeded at a density of 27 million cells in 15 mL media per T75 flask, with 3 flasks seeded per genotype. Effectene Transfection Reagent (Qiagen cat. no. 301427) was used to transfect 3 replicate flasks of cells per genotype. For each replicate, 12 µg plasmid clone library was diluted with Buffer EC to a total volume of 450 µL per flask, 96 µL enhancer was added, and the reaction was vortexed briefly and then incubated for 5 min. One hundred fifty microliters of effectene was added, and the solution was mixed by pipetting up and down 5 times and then incubated for 10 min. One milliliter of media was added to the complex, mixed by pipetting up and down twice, and then added drop-wise onto the flask of cells. Flasks were swirled gently to ensure uniform distribution then returned to 25 °C incubator for 48 h until harvest.

For harvesting, cells were gently pipetted to bring into suspension then centrifuged for 5 min at 350 g. Cells were washed once with 10 mL 1× PBS and then incubated at 37 °C for 5 min in 2 mL M3 + BPYE media containing 1 mL Turbo DNase (Thermo Fisher cat. no. AM2239) per 36 mL. Cells were again pelleted via centrifugation, supernatant removed, and then resuspended in 10 mL 1× PBS. An aliquot of 10% unlysed cells per flask was set aside for later plasmid extraction (pelleted and stored at −20 °C), and the remaining cells were pelleted and lysed in 2 mL RLT (Qiagen RNeasy Midi Kit cat. no. 75144) plus 20 µL 2-mercaptoethanol (Sigma cat. no. 60-24-2) and then frozen at −80 °C.

#### Generation of Input and STARR-seq Libraries

For each of the 6 transfected flasks, both an input library (derived from fragment inserts of plasmid DNA purified from transfected cells) and a STARR library (derived from plasmid-derived mRNA) were generated. Plasmid DNA was purified from 10% of harvested cells per flask using a QIAprep Spin Miniprep Kit (Qiagen cat. no. 27104). Total RNA was extracted from 90% of harvested cells per flask using a Qiagen RNeasy Midi Kit (Qiagen cat. no. 75144). mRNA was isolated from 75 µg total RNA using Dynabeads Oligo(dT)25 (Invitrogen cat. no. 61005), followed by a DNase digestion with Turbo DNaseI (Thermo Fisher cat. no. AM2239). RNA was cleaned with RNAClean XP beads (Beckman Coulter cat. no. A63987) using a 1.8× bead to sample volume ratio and reverse transcribed using SuperScriptIII (Invitrogen cat. no. 18080093) using a gene-specific primer (CTCATCAATGTATCTTATCATGTCTG). cDNA was purified using a 1.8× bead to sample volume of AMPure XP beads (Beckman Coulter cat. no. A63882) and used in a junction PCR to amplify only plasmid-derived mRNA with primers that span a synthetic intron of the pSTARR-seq_fly reporter vector. cDNA was used in this junction PCR with 15 cycles (PCR conditions: initial denaturation of 98 °C for 45 s, then 15 cycles of (i) denaturation: 98 °C for 15 s, (ii) annealing: 65 °C for 30 s, (iii) elongation: 72 °C for 70 s, followed by a final elongation at 72 °C for 60 s) using junction_F (TCGTGAGGCACTGGGCAG*G*T*G*T*C) and junction_R (CTTATCATGTCTGCTCGA*A*G*C) primers and 2× KAPA HiFi HotStart Ready Mix (Roche cat. no. KK2601). Junction PCR products were purified with a 0.8× bead to sample volume ratio with AMPure XP Reagent beads (Beckman Coulter cat. no. A63881). Either 10 ng plasmid DNA (input) or entire cleaned junction PCR products (STARR libraries) were used as input for a PCR to add indices for sequencing, and final libraries were quantified using the dsDNA HS Assay Kit (Invitrogen cat. no. Q32854) on a Qubit4 Fluorometer and average sizes of each library were determined with Agilent High Sensitivity DNA reagents on an Agilent 4200 TapeStation (Agilent cat. nos. 5067-5592, 5067-5593, 5067-5594). Libraries were sequenced on 1 flowcell of a NovaSeq SP with 2 × 50 nt paired-end reads at the Genomics Core Facility at Princeton University. Additional library information and accession numbers are in supplementary table S18, Supplementary Material online.

#### STARR-seq Data Processing and Identification of Enhancers

Raw FASTQ files were processed to remove low-quality bases and adapter contamination using fastp (Chen et al. 2018) with default parameters. Processed FASTQ files were then aligned to the *S. invicta* genome (GCF_016802725.1) with bwa mem (Li 2013) and sorted with SAMtools (Danecek et al. 2021). Enhancer peaks were called with MACS2 (Zhang et al. 2008) on each replicate flask, with input libraries as controls and the following parameters: -f BAMPE and -g 3.8e8 –keep-dup all -q 0.05.

Peaks called on each flask (3 per genotype, 6 total) were concatenated, sorted, and merged with BEDtools (Quinlan and Hall 2010), resulting in a set of 67,105 peaks. This merged set of peaks was used with featureCounts from the Subread package (Liao et al. 2014) to count reads mapping to each peak region for all input and STARR library replicates. The R packages limma (Ritchie et al. 2015) and edgeR (Robinson et al. 2009) were used to identify genomic regions with significant enhancer activity using the following model: ∼genotype + librarytype:genotype. Of the peaks, 23,616 had significant positive enhancer activity in one or both genotypes and were retained. Enhancer peaks were assigned to gene features using the publicly available RefSeq annotation GCF_016802725.1_UNIL_Sinv_3.0_genomic.gff, with TSS flanks defined as the TSS ± 200 bp, promoters defined as 0 to 5 kb upstream of gene start, upstream regions defined as 0 to 10 kb upstream of gene start, and downstream regions defined as 0 to 10 kb downstream of gene start. Exons and introns were extracted from the RefSeq annotation. Peaks that did not overlap any of these features were annotated as intergenic, and peaks that overlapped multiple regions were assigned in the following priority: TSS flank, promoter, intron, upstream, downstream, exon, and intergenic. The function overlapPermTest in the R package regioneR (Gel et al. 2016) was used to assess whether the overlap of peaks with particular features was more than expected by chance.

DAEs between SB and Sb were identified using the R packages limma (Ritchie et al. 2015) and edgeR (Robinson et al. 2009) as in Jones et al. (2024). Peaks were tested for significant interactions between genotype (SB and Sb) and library type (RNA or DNA) using the following model design: ∼genotype + librarytype + genotype*librarytype. The qvalue package (Storey et al. 2024) was used to correct *P*-values for the interaction term, and peaks with q < 0.05 were considered DAEs. For plotting purposes, enhancer strength was quantified as the log2 fold-change of normalized counts from mRNA (STARR libraries) relative to DNA input (input libraries) for each replicate.

### Annotation

#### TF Motif and GO Enrichment

To determine whether specific regulatory motifs are involved in supergene-mediated regulatory variation, we used HOMER (v4.10) (Heinz et al. 2010) and the JASPAR2024 database of insect motifs (Rauluseviciute et al. 2024) to identify enriched TF motifs with ATAC-seq and STARR-seq peaks related to supergene genotype. Specifically, the findMotifsGenome.pl script was used with input files including differential ATAC-seq peaks (DAPs) and differential STARR-seq peaks (DAEs) related to supergene genotype. Results of HOMER and GO for DAPs and DAEs are provided in supplementary tables S5, S6, S14, and S15, Supplementary Material online. GOATools (Klopfenstein et al. 2018) was used for GO analyses of ATAC-seq and STARR-seq peak data sets. Visualization of GO enriched terms by semantic similarity (as in supplementary figs. S5 and S6, Supplementary Material online) was performed using GO-Figure! (Reijnders and Waterhouse 2021).

#### TF Orthologs

To identify a set of putative TFs in the *S. invicta* genome, we performed a 1-way blastp querying the same fire ant genome used for mapping RNA-seq data (GCF_016802725.1) against the latest available *D. melanogaster* genome (GCF_000001215.4). We identified the best hit based on E-value. This approach reduced our set of 14,064 hits to 9,368. We then used FlyBase to identify *D. melanogaster* genes annotated to “DNA-binding transcription factor activity” (GO:0003700). We used these FlyBase IDs to subset the *D. melanogaster* proteins and identify the *S. invicta* genes that encoded the *S. invicta* proteins found as best hits to *D. melanogaster* TF proteins. Of the 555 *D. melanogaster* genes annotated to “DNA-binding transcription factor activity” (GO:0003700), we identified 452 *S. invicta* orthologs which served as our set of putative DNA-binding TFs. For a more inclusive list of TFs, amino acid sequences of all protein-coding genes were first scanned with InterProScan (Jones et al. 2014), and all genes with a significant domain hit to a known DNA binding domain (Zf_C2H2, homeobox_like, HTH, WH, LIM_type, HMG_box, Zf_GATA, Pax, bHLH, BTB_POZ, POU, bZIP, p53_like,Zf_TFIIB_type, MADS_box, Znf_hrmn_rcpt) were treated as putative DNA-binding factors.

#### CNVs

We annotated genes in GCF_016802725.1_UNIL_Sinv_3.0 by their CNV status using a previously published set of CNV transcripts (Fontana et al. 2020). To map transcripts to the current assembly, we used GMAP (Wu and Watanabe 2005) to align all nr-sinv-ref.fasta transcripts and then overlapped predicted exons from GMAP to gene models. Hits with <90% sequence identify were removed, resulting in 25,094 transcripts with unique hits and 1,475 transcripts with multiple hits to 14,192 annotated genes in UNIL 3.0 (*SB*). Next, CNV log2 values (*Sb* vs. *SB*) from supplemental information of Fontana et al. (2020) were used to infer CNV direction and strength for each gene with a significant CNV transcript hit. Because many of the CNV transcripts map to more than 1 locus, the 201 CNV transcripts with at least one >90% identity to exons (of 260 reported CNVs in Fontana et al. 2020) resulted in CNV values for 267 total genes. In addition, some genes had multiple significant transcripts aligning to them; in this case, we selected the highest magnitude log2 fold-change value for that gene when visualizing CNV strength. CNV values for all genes with hits from significant transcripts are provided in supplementary table S7, Supplementary Material online.

#### TEs

In this study, TE annotation was conducted using the Extensive de novo TE Annotator (EDTA) version 2.1.0 (Ou et al. 2019) For the reference genome assembly (GCF_016802725.1_UNIL_Sinv_3.0), the analysis utilized the genome file (GCF_016802725.1_UNIL_ Sinv_3.0_genomic.fna) and its corresponding coding sequences (CDS; GCF_016802725.1_UNIL_Sinv_3.0_ cds_from_genomic.fna). The EDTA analysis was executed with parameters to prevent file overwriting (–overwrite 0), enhance TE detection sensitivity (–sensitive 1), perform TE annotation (–anno 1), evaluate annotation quality (–evaluate 1), and force execution (–force 1). To evaluate TE content in earlier genome builds (GCA_009650705.1_Solenopsis_invicta_SB1.0 and GCA_010367695.1_ASM1036769v1), transcriptomic sequences (rna.fna) from the reference assembly were mapped to these genomes using GMAP-GSNAP v11.3.0 (Wu et al. 2016) with a minimum identity threshold of 90% (–min-identity = 0.90) and options for generating gene-structured GFF3 output (–gff3-fasta-annotation = 1 and -f gff3_gene). The resulting GFF3 files were then refined using the Another Gtf/Gff Analysis Toolkit (AGAT) v1.1.0 (Dainat 2023). In particular, the script agat_sp_fix_features_locations_duplicated.pl was executed with the “–model 1,2,3” option to address various annotation scenarios. For instance, when multiple isoforms exhibited identical exon arrangements, redundant entries were culled by retaining only the transcript with the longest CDS. Similarly, if mRNA entries from distinct gene identifiers had the same exon structure but lacked CDS information, 1 duplicate was removed. In cases where transcripts from different genes had both matching exon and CDS patterns, only the transcript with the longest CDS was preserved. Moreover, when transcripts shared an identical exon structure yet differed in CDS configuration, the tool adjusted the untranslated regions to redefine mRNA and gene boundaries. Lastly, for overlapping mRNA entries with differing exon architectures, AGAT modified gene coordinates by trimming the UTRs. The corrected GFF3 files were subsequently processed using the agat_sp_extract_sequences.pl script to extract refined sequences, which then served as input for EDTA, ensuring a consistent TE annotation process across all genome builds.

#### Variant Calling, Filtering, and Visualization

In this study, we utilized SAMtools v1.16.1 (Danecek et al. 2021) and Picard v2.27.5 (Broad_Institute 2019) in conjunction with GATK v4.4.0.0 (McKenna et al. 2010) for data preprocessing and variant calling. The GATK functions, including HaplotypeCaller with the -ERC GVCF mode, GenomicsDBImport, GenotypeGVCFs, and VariantFiltration, were used to identify and filter variants from both ATAC-seq and STARR-seq data sets. Filtering criteria applied during variant calling included thresholds for depth (DP < 10.0), quality (QUAL < 30.0), quality by depth (QD < 2.0), mapping quality (MQ < 40.0), FisherStrand (FS > 60.0), strand odds ratio (SOR < 3.0), mapping quality rank sum test (MQRankSum < −12.5), and read position rank sum test (ReadPosRankSum < −8.0). Additionally, further filtering to select for biallelic sites was conducted using BCFtools v1.15.1 (Danecek et al. 2021) with the following parameters: -m2 -M2 -v snps. Subsequently, the filtered reads were converted into a variants table using the GATK VariantsToTable function with following parameters: -F CHROM -F POS -F TYPE -F REF -F ALT -GF Ad. Variants were visualized using the VIVA VariantVisualization program (Tollefson et al. 2019), with the following parameters: -x –avg_dp sample,variant –save_remotely.

Previously published raw reads from fire ant samples collected in Georgia (PRJNA182127; Martinez-Ruiz et al. 2020) and Argentina (PRJNA542606; Martinez-Ruiz et al. 2020) were downloaded using the SRA-Toolkit v3.0.1 (fasterq-dump; https://github.com/ncbi/sra-tools/wiki/HowTo:-fasterq-dump). See supplementary table S16, Supplementary Material online for sample details. Adapter removal was performed on the raw FASTQ files using fastp v0.23.2 (Chen et al. 2018), and the trimmed reads were mapped to the reference genome (GCF_016802725.1_UNIL_Sinv_3.0) using BWA v0.7.17 (mem; Li 2013). The resulting alignments were sorted with SAMtools, and variants were subsequently called using GATK with the same parameters described above.

#### Evaluation of SNP Effects on Predicted TF Binding and Enhancer Activity

Fixed or nearly fixed variants between *SB* and *Sb* were determined using the above population genetics data sets including individuals from Argentina and Georgia. Variants of interest were defined as follows for each population: fixed variants had reference allele frequencies of 1 for either *SB* or *Sb* and 0 for the other genotype, while nearly fixed variants had reference allele frequencies of at least 0.8 for either *SB* or *Sb* and less than 0.2 for the other genotype. Of the 283,725 total SNPs called across Argentinian and Georgian populations, 679 were nearly fixed between *SB* and *Sb* in Georgia samples, while 331 SNPs were nearly fixed between *SB* and *Sb* in Argentina samples, with 160 SNPs common to both (supplementary table S19, Supplementary Material online). For completely fixed variants, we identified 573 in Georgia and 183 in Argentina between *SB* and *Sb* individuals, 60 of which were common to both (supplementary table S19, Supplementary Material online).

For each SNP, 2 101 bp regions (one for each allele) centered on the SNP of interest were scanned with FIMO (Grant et al. 2011) using the JASPAR2024 database of nonredundant insect motifs (Rauluseviciute et al. 2024). A SNP was considered to have an effect on predicted binding for a given motif if only 1 allele had a significant (*P* < 0.0001) match for that motif and/or if the ratio of FIMO scores between the 2 alleles was >1.5, as in Kapheim et al. (2020) and Jones et al. (2024). Results of FIMO are provided in supplementary table S17, Supplementary Material online.

## Supplementary Material

msaf112_Supplementary_Data

## Data Availability

Sequencing data are deposited in NCBI's Sequencing Read Archive under accession PRJNA1071646.
